# Production, partial purification and characterization of ligninolytic enzymes from selected basidiomycetes mushroom fungi

**DOI:** 10.1016/j.sjbs.2021.08.026

**Published:** 2021-08-13

**Authors:** Ramanaiah Illuri, M. Kumar, M. Eyini, V. Veeramanikandan, Khalid S Almaary, Yahya B. Elbadawi, M.A. Biraqdar, P. Balaji

**Affiliations:** a PG and Research Centre in Biotechnology, MGR College, Hosur, Tamil Nadu, India; b Department of Plant Biology and Plant Biotechnology, Madras Christian College (Autonomous), Tambaram, Chennai, Tamil Nadu, India; c Department of Botany, Thiagarajar College (Autonomous), Madurai, Tamil Nadu, India; d PG and Research Centre in Microbiology, MGR College, Hosur, Tamil Nadu, India; e Department of Botany and Microbiology, College of Science, King Saud University, P.O. 2455, Riyadh 11451, Saudi Arabia; f College of Biological Sciences, The University of Western Australia, 35 Stirling Highway, Perth, WA 6009 Australia

**Keywords:** Ligninolytic enzymes, SDS-PAGE, Enzyme purification, Enzyme activity, Basidiomycete fungus

## Abstract

In recent years, many research on the quantity of lignocellulosic waste have been developed. The production, partial purification, and characterisation of ligninolytic enzymes from various fungi are described in this work. On the 21st day of incubation in Potato Dextrose (PD) broth, *Hypsizygus ulmarius* developed the most laccase (14.83 × 10^−6^ IU/ml) and manganese peroxidase (24.11 × 10^−6^ IU/ml), while *Pleurotus florida* produced the most lignin peroxidase (19.56 × ^−6^ IU/ml). Laccase (Lac), lignin peroxidase (LiP), and manganese peroxidase (MnP), all generated by selected basidiomycetes mushroom fungi, were largely isolated using ammonium sulphate precipitation followed by dialysis. Laccase, lignin peroxidase, and manganese peroxidase purification findings indicated 1.83, 2.13, and 1.77 fold purity enhancements, respectively. Specific activity of purified laccase enzyme preparations ranged from 305.80 to 376.85 IU/mg, purified lignin peroxidase from 258.51 to 336.95 IU/mg, and purified manganese peroxidase from 253.45 to 529.34 IU/mg. *H. ulmarius* laccase (376.85 IU/mg) with 1.83 fold purification had the highest specific activity of all the ligninolytic enzymes studied, followed by 2.13 fold purification in lignin peroxidase (350.57 IU/mg) and manganese peroxidase (529.34 IU/mg) with 1.77-fold purification. Three notable bands with molecular weights ranging from 43 to 68 kDa and a single prominent band with a molecular weight of 97.4 kDa were identified on a Native PAGE gel from mycelial proteins of selected mushroom fungus. The SDS PAGE profiles of the mycelial proteins from the selected mushroom fungus were similar to the native PAGE. All three partially purified ligninolytic isozymes display three bands in native gel electrophoresis, with only one prominent band in enzyme activity staining. The 43 kDa, 55 kDa, and 68 kDa protein bands correspond to laccase, lignin peroxidase, and manganese peroxidase, respectively.

## 1 Introduction

Concerns about pollution and environmental conservation have compelled us to look for a new generation of cleaner industrial production that maximises efficiency while minimising contamination. In addition to cellulose and hemicellulose, microorganisms, especially those of basidiomycetes phylum, were able to degrade lignin effectively. Based on their wood-decaying patterns, these basidiomycetes fungi are classified as white, brown, or soft rot fungi. White rot fungus appear to be well-known for their capacity to quickly degrade lignin, while the other two digest plant biomass components quickly but take longer to decompose lignin (Kameshwar and Qin, 2017).

Extracellular enzymatic systems discovered in white-rot fungus include the hydrolytic system, which produces hydrolases necessary for polysaccharide breakdown, and a distinct reactive and extrinsic ligninolytic system, which breaks lignin and opens phenyl rings. They naturally produce several lignin-degrading extracellular enzymes, including copper containing laccase, heme containing manganese peroxidase, and lignin peroxidase (LiP), as well as aryl alcohol oxidase (AAO), and major hydrolytic enzymes such as amylases and xylanase. In a extensive variability of biotechnology applications, together with paper, food, textiles, the colouring industry, bioremediation, cosmetics, etc., the white-red fungal enzymes are essential to the efficient conversion of plant residues (Ergun and Urek, 2017, Lundell et al., 2010)

Lignin, assumed to be the most plentiful and composite biopolymer due to its low biodegradability. The recalcitrant compound of lignocellulosic materials results from the complex chemical bonding among its subunits. Phenylpropane groups are formed by radical polymerizing guaiacyl, syringyl, and p-hydroxyphenyl components from precursors including coniferyl, p-coumaryl alcohol and sinapyl. Lignin is an aromatic polymer with a high molecular weight and many physiologically stable ether or ester bonds. As a result, cellulose fibres become entangled in a complex mixture of hemicellulose and lignin, preventing cellulases and hemicellulases from working. Owing to the difficulty of lignocellulosic waste degradation, it poses a significant challenge to supportable growth. The quantity of these waste generated by several agro-based enterprises, such as paper and pulp industries, distilleries, crop residues, and the food industrial waste, demonstrates the reach of the issue. Due to their deliberate biodegradation and enzymatic activities with other cationic substances, they form composite toxic substances. As a consequence, lignocellulosic product that contains toxic waste contributes contributes to global pollution (Sadh et al., 2018, Taha et al., 2016, Ravindran and Jaiswal, 2015).

Ligninolytic enzymes are essential for lignocellulosic waste degradation and detoxification in the environment. The most common ligninolytic enzymes are Lac, LiP, MnP, and versatile peroxidase. The activity of those enzymes in the breakdown and digestion of lignocellulosic waste is enhanced by a range of intermediaries and other enzymes such as aryl-alcohol oxidase, catechol 2, 3-dioxygenase, feruloyl esterase, lipases, quinone reductases. The primary objective of this study is to describe and partially purify fungal extracellular ligninolytic enzymes. Experiments were intended to examine only extracellular enzymes, the majority of which are ligninolytic in nature. The results provided in this article are the first to demonstrate the extracellular ligninolytic enzymes of 15 basidiomycetes mushroom fungi. The enzymatic characteristics of ligninolytic enzymes from fifteen edible basidiomycetes mushroom fungi were investigated in this research. The current research adds to our understanding of the biochemical properties of ligninolytic enzymes found in mushroom fungi.

## 2 Materials and methods

### 2.1 Basidiomycete fungi

The pure cultures of mushroom fungus strains utilised for the research are listed along with the source and their strain numbers in Table 1.

**Table 1:** Binomials, strain numbers, and edible basidiomycetes mushroom fungi sources.

Mushroom Fungi	Common Name	Source	Strain No.
***Pleurotus sajor-caju*** (Fr.) Singer	Oyster Mushroom	TNAU	M_2_
***Pleurotus djamor*** (Fr.) Boedijn	Oyster Mushroom	TNAU	MDU_1_
***Pleurotus citrinopileatus*** Singer	Oyster Mushroom	TNAU	CO_1_
***Pleurotus eous*** (Berk.) Sacc.	Oyster Mushroom	TNAU	APK_1_
***Pleurotus cystidiosus*** (OK) Miller	Oyster Mushroom	UM	Wild Isolate
***Pleurotus ostreatus*** (Jacq. ex Fr.) Kumm.	Oyster Mushroom	UM	Wild Isolate
***Pleurotus flabellatus*** (Berk and Br.) Sacc.	Oyster Mushroom	TNAU	MDU_2_
***Pleurotus florida*** (Mont.) Singer	Oyster Mushroom	TNAU	PF
***Pleurotus pulmonarius*** (Fr.) Quelet.	Usushiratake	MRIJ	PP
***Hypsizygus ulmarius*** (Bull.:Fr.) Redhead	Elm Oyster	UM	Wild Isolate
***Oudemansiella radicata*** (Relhan ex Fr.) Sing.	Rooted Collybia	UM	Wild Isolate
***Volvariella volvacea* (**Bulliard ex Fries) Sing.	Paddy Straw Mushroom	UM	Wild Isolate
***Schizophyllum commune*** Fries	Split Gill	UM	Wild Isolate
***Calocybe indica*** Purkayastha & A. Chandra	Milky Mushroom	TNAU	APK_2_
***Tricholomopsis giganteus***	Giant polypore	UM	Wild Isolate

TNAU – Tamil Nadu Agricultural University, Coimbatore, India.

UM – University of Madras, Guindy Campus, Chennai, India.

MRIJ – Mushroom Research Institute, Japan.

### 2.2 Maintenance of fungal basidiomycetes

Species of pure mushrooms were stored at 10 °C. Subculture was performed every month to check the species remains viable. The mycelial agar plugs from stock cultures (7 mm) were aseptically transferred into fresh potato dextrose agar (PDA) medium and kept at 28 ± 2 °C to achieve a confluence of growth.

### 2.3 Linear downward growth and radial growth

The Linear Downward Growth (LDG) of basidiomycetes mushroom fungi and their mycelial proliferation on unfilled paddy grain substrate were examined using Rafique (1998) procedure with minor modifications. Unfilled paddy grains were soaked in water overnight, drained, and the substrate was filled in boiling tubes to a length of 10 cm with a 60% moisture content. Single agar blocks (8 mm diameter) of seven-day-old culture of the chosen basidiomycetes mushroom fungi were aseptically inoculated into the individual tubes after sterilisation. The tubes were incubated at 28 ± 2 °C for the time it took for linear downward growth of various fungi to reach the bottom of the tube, as well as the degree of mycelial proliferation as measured by mycelial biomass, which was classified as good, moderate, dense, or low.

For radial growth assessment, mycelial agar blocks (8 mm diameter) were cut from the margins of seven-day-old fungal colonies and inoculated singly in the centre of culture plates containing PDA medium. The culture plates were kept undisturbed in incubator at 28 ± 2 °C for seven days. The mycelial radius after seven days of growth in petridish was used to determine radial growth (Soccol et al., 1994).

### 2.4 Biomass production and protein determination

From the growing ends of seven days old culture, single agar plugs of 8 mm diameter containing mycelium of different mushroom fungi were transferred to sterilized 50 ml of Potato Dextrose Broth (PDB). The flasks were cultured as static cultures for 21 days at 28 ± 2 °C to collect the mycelial biomass. The fugal biomass was extracted from the culture medium using vacuum filtering with Whatman filter paper. After that, the biomass was rinsed with sterile Milli-Q water, blotted dry, measured, and used for further research. The protein content of the mycelial biomass was calculated using Bradford (1976) method with minor modifications. In 5 ml of Tris-HCl buffer, fresh mycelium (1 g) was added (pH 6.8). For 10 min, the mixture was held at 4 °C. The mixture was then homogenised with a pestle in a mortar. The homogenate was centrifuged for 5 min at 3000 rpm. The protein content of the supernatant was calculated. Using a standard graph prepared with Bovine Serum Albumin protein, the amount of protein in the sample was measured.

### 2.5 Qualitative plate assays

#### 2.5.1 Ligninase

Basal medium containing lignin (1.6%) was sterilized and then incorporated individually with sterilized 1 ml of each of glucose (20%) and tannic acid (1%). After aseptically transferring the medium to petri plates, a single mycelial agar plug is inoculated from seven-day-old mushroom fungal cultures and incubated in dark at 28 °C. The presence of a brown oxidisation area around the fungal colonies is represented by total polyphenol oxidase activity. The diameter of the brown zone around the colony was investigated and measured in the Petridishes. To determine the production potential of ligninase in fungi, the zone diameter/colony diameter (Z/C) ratio was determined (Kameshwar and Qin, 2017)

#### 2.5.2 Polyphenol oxidase

Erlenmeyer flask was used to prepare the basal medium (90 ml). Green tea extract was made by boiling tea leaves in water for 10 min at a concentration of 20% w/v. The extract was mixed into the modified basal medium, which had a pH of 4.5. After autoclaving for 15 min, the flasks were cooled and dispensed in sterile petri plates. The subsequent rate of quinone production, as revealed by an rise in absorbance units (AUs) at 420 nm, was used to evaluate polyphenol oxidase activity (Taranto et al., 2017).

#### 2.5.3 Laccase

By inoculating 1 cm wide mycelium from every strain onto Crawford's improved medium (100 ml) and incubated at 28 °C, filamentous fungi were evaluated for primary laccase activity. 2 mM guiacol was dispensed in sterile petri plates after the contents of the flask been autoclaved for 15 min and chilled. A single mushroom fungal agar block (7 mm) was inoculated from a seven days old culture that was actively growing. The reddish brown area around the colony indicates laccase enzyme production (Góralczyk-Bińkowska et al., 2020).

### 2.6 Quantitative enzyme activity assays

#### 2.6.1 Laccase

Laccase activity was assessed using guaiacol as a medium. The reaction mixture consists of 3 ml of 100 mM guaiacol dissolved in 10% acetone in sodium acetate buffer and 1 ml of culture filtrate (crude laccase). The absorbance at 470 sample was measured with a UV–visible spectrophotometer following 15 min of incubation, and the enzyme activity was expressed in International Units per millilitre (IU/ml). Blanks were maintained with sterile water (Senthivelan et al., 2019).

#### 2.6.2 Lignin peroxidases

The activity of Lignin Peroxidase was tested by oxidising veratryl alcohol. The assay included 900 µl sodium tartrate buffer (25 mM; pH 2.5), 0.4 mM hydrogen peroxide, 25 mM veratryl alcohol, and 100 µl enzyme source. After 1 min of room temperature incubation, the absorption spectrum of the test sample was measured at 310 nm using a UV–visible spectrophotometer, and the activity of the enzyme was expressed in IU/ml. Assay blanks is made up of distilled water (Nayak et al., 2020).

#### 2.6.3 Manganese peroxidase

Using hydrogen peroxide oxidation, the performance of manganese peroxidases was examined. 900 µl sodium tartrate buffer (100 mM; pH 5) with 0.1 mM MnSO_4_, 0.1 mM H_2_O_2_, and 100 µl enzyme source were used in the test. The assay material was kept at room temperature for 1 min until being quantified in IU/ml using a UV–visible spectrophotometer at 238 nm. A change in absorption spectrum of 0.01 min^−1^ was equal to one unit of enzyme activity. Throughout the test, sterile water was used to maintain the blanks (Lueangjaroenkit et al., 2019).

### 2.7 Screening of selected mushroom fungi for partially purified extracellular ligninolytic enzyme activities

#### 2.7.1 Partial purification of extracellular ligninolytic enzymes

According to Elias et al., 2000, Vares et al., 1995 the mushroom cultures ligninolytic enzymes were partly purified. Basal medium (100 ml) was prepared in a 250 ml Erlenmeyer flask, inoculated with basidiomycetes mushroom fungi, and incubated for 21 days at 28 °C. The medium was centrifugated at 7800 g for 20 min. The supernatant (culture filtrate) obtained after centrifugation was used as an enzyme source. To facilitate the precipitation of extracellular polysaccharides, the culture filtrate that was collected by centrifugation was held at −4°C overnight. The volume of the supernatant was quantified, and 1000 μl of each sample was frozen at −20 °C for use in an enzyme purification graph to quantify protein and ligninolytic enzyme activity.

#### 2.7.2 Ammonium sulphate precipitation

To ensure thorough precipitation, an ammonium sulphate solution was then added to the culture supernatant and equilibrated at 4 °C for 30 min. The precipitates were extracted by centrifugation at 10,000g for 10 min at 4 °C, then redissolved in 2.5 ml sodium acetate buffer (25 mM, pH 5.5), and the protein content and enzyme activity were measured at each step. The active fractions that precipitated at a saturation of 80% ammonium sulphate were combined and used for dialysis purification (Ajith et al., 2019).

#### 2.7.3 Purification by dialysis

The samples were dialyzed at 4 °C against a 25 mM sodium acetate buffer using a dialysis membrane with a molecular weight cut-off of 10,000 Dalton (pH 5.5). The samples were stirred for 12 h at 4 °C on a mechanical stirring after being immersed in a 50-fold volume of 25 mM sodium acetate buffer (pH 5.5). During the incubation time, fresh buffer was replaced every 4 h. The volume of the dialysates was measured, and 500 µl of dialysate samples were used to calculate protein and ligninolytic enzyme activities (Gaonkar and Furtado, 2020).

#### 2.7.4 Protein estimation and electrophoresis

Using BSA as a reference, the concentration of protein in the sample was measured using Bradford (1976) technique. Ligninolytic enzymes of the mushroom cultures were partially purified following the method of Elias et al., 2000, Vares et al., 1995. The purification level of ligninolytic enzyme in each purification step was calculated as described by Dennison (2002). SDS-PAGE is used to separate proteins mostly under reducing conditions, a 12% separating gel (pH 8.8) and a 5% stacking gel (pH 6.8) were performed to measure the subunit molecular weight of the recovered ligninolytic enzyme, as previously stated by Laemmli (1970). The isolated ligninolytic enzyme was run in Native-PAGE on a 12% resolving gel in Tris-glycine buffer (pH 8.5) at 4 °C. By comparing pure ligninolytic protein to standard protein markers, the molecular mass of the protein was calculated. Coomassie Brilliant Blue R-250 staining was used to visualise the protein bands (Fijałkowska et al., 2020, Benmrad et al., 2019, Cicatiello et al., 2019). For Laccase, LiP, and MnP activity staining, electrophoresed gels (Native PAGE) were separated and washed with sodium tartrate buffer. The laccase zymogram gel was soaked in a solution containing sodium tartrate buffer (25 mM; pH 2.5), veratryl alcohol (25 mM), and hydrogen peroxide (H_2_O_2_), while the LiP zymogram gel was soaked in a solution containing sodium tartrate buffer (25 mM; pH 2.5), veratryl alcohol (25 mM), and hydrogen peroxide (0.4 mM). MnP enzyme gels were soaked in a solution containing 100 mM sodium tartrate buffer (pH 5), phenol red (0.1%), MnSO_4_ (1 mM), and hydrogen peroxide (0.1 mM) (Schneider et al., 2019, Schulze et al., 2019).

## 3 Results

### 3.1 Linear downward growth

On unfilled paddy grains, linear downward growth and mycelial proliferation of fifteen mushroom fungi were examined, including nine *Pleurotus* (oyster mushroom) species, one species from each genus of *Hypsizygus, Oudemansiella, Volvariella, Schizophyllum, Tricholomopsis*, and *Calocybe*. *Pleurotus eous, Pleurotus sajor-caju, Pleurotus flabellatus, Pleurotus djamor, Pleurotus pulmonarius, Schizophyllum commune*, and *Volvariella volvacea* grew faster linearly downward on unfilled paddy grain substrate than the other mushrooms, taking 12–15 days to develop a 10 cm length of substrate. Under the same conditions, *Hypsizygus ulmarius, P. ostreatus, P. citrinopileatus, P. florida*, and *P. cystidiosus* colonised the same length of substrate in 16–17 days*.*

*Calocybe indica, Oudemansiella radicata*, and *Tricholomopsis giganteus* showed poor linear downward development, colonising the 10 cm length of substrate in a maximum of 21–26 days. Except for *P. eous, V. volvacea*, and *S. commune*, all of the selected mushroom species provided dense biomass on unfilled paddy grains substrate, while the latter displayed sparse mycelial density on the same substrate (Table 2).

**Table 2:** Linear downward growth and mycelial density of different basidiomycetes mushroom fungi on unfilled paddy grain.

S. No.	Mushroom fungi	Linear downward growth (days)	Mycelial density
1.	*Pleurotus sajor-caju*	14 ± 0.3	+++
2.	*P. djamor*	15 ± 0.6	+++
3.	*P. citrinopileatus*	17 ± 0.3	+++
4.	*P. eous*	13 ± 0.5	++
5.	*P. cystidiosus*	18 ± 0.4	+++
6.	*P. ostreatus*	17 ± 0.2	+++
7.	*P. florida*	14 ± 0.3	+++
8.	*P. flabellatus*	17 ± 0.5	+++
9.	*P. pulmonarius*	15 ± 0.7	+++
10.	*Hypsizygus ulmarius*	16 ± 0.5	+++
11.	*Oudemansiella radicata*	24 ± 0.3	+++
12.	*Volvariella volvacea*	13 ± 0.1	++
13.	*Schizophyllum commune*	12 ± 0.5	++
14.	*Tricholomopsis giganteus*	26 ± 0.8	+++
15.	*Calocybe indica*	21 ± 0.6	+++

++: Sparse +++: Dense.

### 3.2 Radial growth

The radial growth and growth rate of the selected mushroom fungi on potato dextrose agar medium are shown in Table 3. The fifteen mushroom fungi studied showed a broad range of seventh-day radial development, ranging from 1.9 to 3.9 cm.

**Table 3:** Radial growth of different mushroom fungi on potato dextrose agar medium on 7^th^ day of incubation.

S. No.	Mushroom fungi	Radial growth (cm)
1.	*Pleurotus sajor-caju*	2.95 ± 0.08
2.	*P. djamor*	2.60 ± 0.03
3.	*P. citrinopileatus*	2.60 ± 0.04
4.	*P. eous*	3.50 ± 0.03
5.	*P. cystidiosus*	1.90 ± 0.12
6.	*P. ostreatus*	2.10 ± 0.01
7.	*P. florida*	2.60 ± 0.02
8.	*P. flabellatus*	2.40 ± 0.01
9.	*P. pulmonarius*	3.15 ± 0.02
10.	*Hypsizygus ulmarius*	3.10 ± 0.04
11.	*Oudemansiella radicata*	1.95 ± 0.04
12.	*Volvariella volvacea*	3.90 ± 0.01
13.	*Schizophyllum commune*	2.30 ± 0.10
14.	*Tricholomopsis giganteus*	2.00 ± 0.06
15.	*Calocybe indica*	2.70 ± 0.01

On the seventh day of incubation, *Volvariella volvacea* (3.9 cm), *Pleurotus eous* (3.5 cm), *Pleurotus pulmonarius* (3.15 cm), and *H. ulmarius* (3.1 cm) grew faster than the other mushrooms on potato dextrose agar medium. *P. sajor-caju* (2.95 cm), *C. indica* (2.7 cm), *P. florida* (2.6 cm), *P. djamor* (2.6 cm), *P. citrinopileatus* (2.6 cm), *P. flabellatus* (2.4 cm), *S. commune* (2.3 cm), *P. ostreatus* (2.1 cm), and *T. giganteus* all showed moderate radial development (2.0 cm). On the seventh day of incubation, two of the fifteen mushroom gerera, *O. radicata* (1.95 mm) and *P. cystidiosus* (1.9 cm), showed very slow development.

*V. volvacea* and *P. eous* colonised the entire petriplate containing PDA medium in 8 and 9 days, respectively, while *P. pulmonarius, H. ulmarius*, and *P. sajor-caju* took 10–11 days to cover the entire plate. *P. cystidiosus*, *O. radicata*, and *T. giganteus* grew at a much slower pace, colonising the entire plate in 17 or 16 days.

### 3.3 Biomass and mycelial protein

Table 4 shows the mycelial biomass and mycelial protein content of the selected mushroom fungi on the 21^st^ day of PD broth growth. Among the selected mushroom fungi, *S. commune* had the highest mycelial fresh weight (5.39 g) in PD broth, but the lowest mycelial protein content (0.87 mg). The protein content of *P. ostreatus* was the lowest. *H. ulmarius* (5.22 g), *P. djamor* (4.88 g), *P. cystidiosus* (4.49 g), and *O. radicata* had the lowest mycelial biomass. Among the fifteen mushrooms studied, *H. ulmarius* had the highest mycelial protein content (2.62 mg/g). *P. eous* (2.56 mg/g), *P. djamor* (2.51 mg/g), *P. florida* (2.40 mg/g), and *P. flabellatus* (2.13 mg/g) had the lowest mycelial protein content. All of the other strains had significantly lower mycelial biomass and protein content than the strains mentioned above.

**Table 4:** Mycelial fresh weight (g) and mycelial protein (mg / g) of 21 days-old cultures of selected mushroom fungi growing on potato dextrose broth.

S. No.	Mushroom fungi	Mycelial Biomass (g)	Mycelial Protein (mg/g)
1.	*Pleurotus sajor-caju*	2.94 ± 0.02	1.85 ± 0.01
2.	*P. djamor*	4.88 ± 0.02	2.50 ± 0.02
3.	*P. citrinopileatus*	3.09 ± 0.01	1.14 ± 0.01
4.	*P. eous*	1.79 ± 0.02	2.56 ± 0.04
5.	*P. cystidiosis*	4.49 ± 0.03	1.69 ± 0.00
6.	*P. ostreatus*	4.38 ± 0.02	0.65 ± 0.01
7.	*P. florida*	4.44 ± 0.01	2.39 ± 0.01
8.	*P. flabellatus*	4.20 ± 0.03	2.12 ± 0.03
9.	*P. pulmonarius*	2.71 ± 0.01	1.58 ± 0.01
10.	*Hypsizygus ulmarius*	5.22 ± 0.02	2.61 ± 0.01
11.	*Oudemansiella radicata*	4.46 ± 0.02	1.79 ± 0.02
12.	*Volvariella volvacea*	3.78 ± 0.03	1.58 ± 0.02
13.	*Schizophyllum commune*	5.39 ± 0.03	0.87 ± 0.01
14.	*Tricholomopsis giganteus*	3.04 ± 0.02	1.30 ± 0.01
15.	*Calocybe indica*	2.60 ± 0.03	1.69 ± 0.03

### 3.4 Qualitative plate assays

#### 3.4.1 Ligninase positive character

The growth of mushroom colony and the formation of a brown zone in tannic acid medium around it indicate the ability of microorganisms to produce the ligninase enzyme. Except for *Volvariella volvacea* and *Tricholomopsis giganteus*, all basidiomycetes mushroom fungi tested positive for ligninase activity on tannic acid plates. Of all the strains analysed, *Hypsizygus ulmarius* and *Oudemansiella radicata* had the highest ligninase activity (brown zone diameter, 4.8 cm), followed by *S. commune* (4.5 cm), *P. florida* (4.2 cm), and *P. djamor* (3.0 cm). Ligninase activity was moderate in *P. cystidiosus* (2.9 cm) and *P. pulmonarius* (2.5 cm). On Tannic acid medium, all of the other strains had low ligninase activity as measured by brown zone diameter. The highest ratio of zone diameter to colony diameter (Z/C), which indicates the efficiency of ligninase production in relation to colony growth, was found for *H. ulmarius* (Z/C – 1.25), accompanied by *P. djamor* (Z/C – 1.15). In terms of Z/C ratio, *O. radicata*, which had the maximum brown region, was the least productive of the organisms tested (Table 5).

**Table 5:** Plate assays for ligninase, polyphenol oxidase and laccase activities of different mushroom fungi.

S. No.	Mushroom fungi	TAM (cm dia)	Z/C	GTM (cm dia)	Z/C	CMM (cm dia)	Z/C
1.	*Pleurotus sajor-caju*	2.6 ± 0.2	1.08 ± 0.03	7.5 ± 0.6	0.97 ± 0.01	7.7 ± 0.5	0.97 ± 0.09
2.	*P. djamor*	3.0 ± 0.4	1.15 ± 0.02	6.2 ± 0.5	1.03 ± 0.03	8.2 ± 0.5	0.96 ± 0.01
3.	*P. citrinopileatus*	1.4 ± 0.2	1.0 ± 0.02	4.0 ± 0.3	0.95 ± 0.02	4.8 ± 0.3	0.96 ± 0.03
4.	*P. eous*	1.2 ± 0.1	1.04 ± 0.01	4.2 ± 0.4	0.93 ± 0.02	6.0 ± 0.4	0.95 ± 0.05
5.	*P. cystidiosis*	2.9 ± 0.4	1.03 ± 0.03	4.2 ± 0.3	0.95 ± 0.02	4.6 ± 0.3	0.95 ± 0.03
6.	*P. ostreatus*	2.1 ± 0.3	0.91 ± 0.03	5.0 ± 0.5	0.96 ± 0.03	5.5 ± 0.4	0.94 ± 0.03
7.	*P. florida*	4.2 ± 0.6	1.31 ± 0.02	5.9 ± 0.6	1.05 ± 0.01	6.2 ± 0.4	0.96 ± 0.02
8.	*P. flabellatus*	2.0 ± 0.3	0.95 ± 0.04	5.4 ± 0.5	0.98 ± 0.07	5.7 ± 0.3	0.95 ± 0.06
9.	*P. pulmonarius*	2.5 ± 0.3	0.92 ± 0.06	4.7 ± 0.4	0.94 ± 0.01	4.2 ± 0.2	0.93 ± 0.04
10.	*Hypsizygus ulmarius*	4.8 ± 0.7	1.26 ± 0.01	5.7 ± 0.5	1.05 ± 0.04	5.6 ± 0.3	0.93 ± 0.01
11.	*Oudemansiella radicata*	4.8 ± 0.5	1.04 ± 0.05	6.2 ± 0.6	0.98 ± 0.05	5.0 ± 0.3	0.94 ± 0.05
12.	*Volvariella volvacea*	No Growth	–	3.5 ± 0.3	0.94 ± 0.02	4.1 ± 0.2	0.93 ± 0.01
13.	*Schizophyllum commune*	4.5 ± 0.5	1.1 ± 0.01	7.0 ± 0.6	0.94 ± 0.02	6.7 ± 0.4	0.96 ± 0.03
14.	*Tricholomopsis giganteus*	No Growth	–	2.6 ± 0.4	0.86 ± 0.01	3.1 ± 0.2	0.91 ± 0.00
15.	*Calocybe indica*	1.7 ± 0.2	1.06 ± 0.00	5.1 ± 0.7	0.92 ± 0.03	4.5 ± 0.3	0.96 ± 0.02

Z/C: Zone diameter/Colony diameter / TAM: Tannic acid medium, GTM: Green Tea Medium; CMM: Crawford’s modified medium

#### 3.4.2 Polyphenol oxidase production

Two types of ligninolytic enzymes are recognised based on their mechanism of action: Peroxidases and polyphenol oxidases. The main polyphenol oxidases are laccase and catechol oxidases. In terms of brown zone diameter, Table 5 indicates the polyphenol oxidase development capacity of various mushrooms.

Plates colonised by *P. sajor-caju* (7.5 cm), *S. commune* (7.0 cm), *P. djamor* (6.2 cm), *O. radicata* (6.2 cm), *P. florida* (5.9 cm), and *H. ulmarius* (5.7 cm) had the largest brown zone on the reverse of the plates containing green tea medium, suggesting the mushroom polyphenol oxidase development capacity. Among the mushroom fungi studied, *T. giganteus* formed the smallest brown zone (2.6 cm). *P. florida* and *H. ulmarius* had the highest efficiency of polyphenol oxidase output determined by Z/C ratio in green tea medium (Z/C – 1.05), indicating that the enzyme output capacity was out of proportion to the colony size. Lower Z/C values (0.98) were found in *P. djamor* and *O. radicata*, which formed slightly larger brown zones (6.2 cm) (Table 5).

#### 3.4.3 Laccase assay

On guiacol amended Crawford's modified medium, laccase activity is visualised and measured as the diameter of the reddish brown zone (Table 5). *P. djamor* had the most laccase activity (reddish brown zone diameter, 8.2 cm), followed by *P. sajor-caju* (7.7 cm), *S. commune* (6.7 cm), *P. florida* (6.2 cm), *P. eous* (6.0 cm), *P. flabellatus* (5.7 cm), *H. ulmarius* (5.6 cm), and *T. giganteus* (3.1 cm).

### 3.5 Quantitative enzyme activity assays

**Fig. 1:**
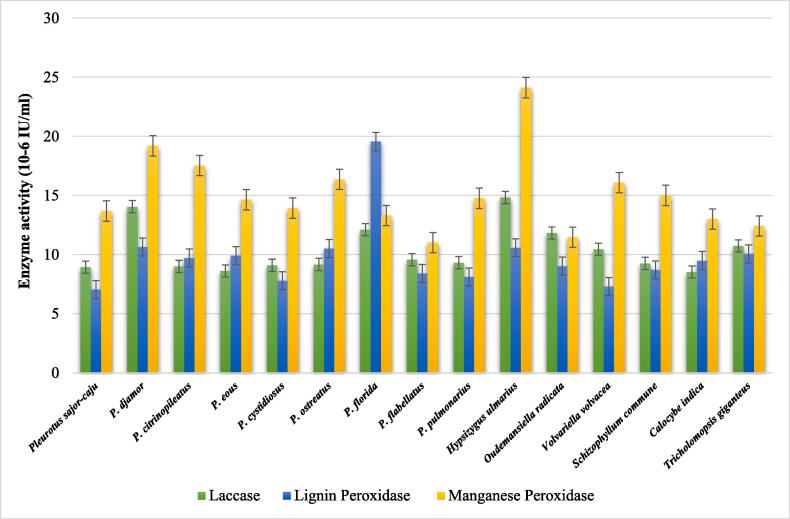
Extracellular ligninolytic enzyme activities of selected mushroom fungi on the 21^st^ day of incubation.

On the 21^st^ day of incubation in PD broth, the extracellular ligninolytic enzymatic activity of the fifteen mushroom fungi were determined and recorded in Fig. 1.

#### 3.5.1 Laccase activity

Laccase activities were significantly higher in *H. ulmarius* (14.83 × 10^−6^ IU/ml) and *P. djamor* (14.05 × 10^−6^ IU/ml), followed by *P. florida* (12.11 × 10^−6^ IU/ml), *O. radicata* (11.83 × 10^−6^ IU/ml), *T. giganteus* (10.73 × 10^−6^ IU/ml), and *V. volvacea* (10.45 × 10^−6^ IU). The laccase activities of the other *Pleurotus* species, namely *P. sajor-caju, P. citrinopileatus, P. eous, P. cystidiosus, P. ostreatus, P. flabellatus, S. commune*, and *C. indica*, ranged between 8.54 and 9.57 × 10^−6^ IU/ml, which were 25% lower than the highest laccase producers (Fig. 1).

#### 3.5.2 Lignin peroxidase activity

Of all the mushroom fungi tested, *P. florida* had the highest LiP activity (19.56 × 10^−6^ IU/ml). *P. djamor, H. ulmarius, P. ostreatus*, and *T. giganteus* had lignin peroxidase activities of 10.06 to 10.64 × 10^−6^ IU/ml, which were substantially lower than *P. florida*. The LiP activities of *V. volvacea* and *P. sajor-caju* were 7.31 × 10^−6^ IU/ml and 7.05 × 10^−6^ IU/ml, respectively (Fig. 1).

#### 3.5.3 Manganese peroxidases activity

Among the fifteen mushrooms tested, *H. ulmarius* had the highest MnP activity (24.11 × 10^−6^ IU/ml), followed by *P. djamor* (19.19 × 10^−6^ IU/ml), *P. citrinopileatus* (17.53 × 10^−6^ IU/ml), *P. ostreatus* (16.36 × 10^−6^ IU/ml), and *V. volvacea* (16.08 × 10^−6^ IU/ml). MnP activity (15.01 × 10^−6^ IU/ml) was significantly higher in *S. commune* with low laccase and LiP activity. Among the mushroom fungi tested, *P. flabellatus* (11.01 × 10^−6^ IU/ml) and *O. radicata* (11.47 × 10^−6^ IU/ml) had the lowest MnP activity (Fig. 1).

### 3.6 Purification of ligninolytic enzyme

The results of the purification procedure are summarized for laccase, lignin peroxidase and manganese peroxidase in Table 6, Table 7, Table 8 respectively. Purified laccase enzyme preparations had specific activity ranging from 305.80 to 376.85 IU/mg, with *H. ulmarius* having the highest specific activity of 376.85 IU/mg with 1.83 fold purification (Table 6), while purified lignin peroxidase had specific activity ranging from 258.51 to 336.95 IU/mg, with 2.13 fold purification in *H. ulmarius* (Table 7). Purified manganese peroxidase had a specific activity of 253.45 to 529.34 IU/mg. With 529.34 IU/mg, *H. ulmarius* was the maximum of all the strains verified, followed by *S. commune* (520.99 IU/mg) and *P. florida* (511.65 IU/mg). With a purification fold of 1.77, *S. commune* had the highest manganese peroxidase enzyme purification (Table 8).

**Table 6:** Partial purification of Laccase from selected mushroom fungi (Results are mean of three replicates with SE < 5%).

Organisms	Purification steps	Volume (ml)	Total protein (mg)	Enzyme activity (×10^−5^ IU/ml)	Total activity (×10^−5^ IU)	Specific activity (×10^−5^ IU/mg)	Yield (%)	Purification (fold)
*Pleurotus djamor*	After 1st Centrifugation	84	387.80	0.94	78.62	202.74	100	1
	After 2nd Centrifugation	80	374.26	0.98	78.56	209.90	99.92	1.03
	Ammonium sulphate precipitation	4	10.99	1.05	7.20	382.52	5.34	1.88
	Dialysis	2	6.70	1.06	2.11	315.49	2.68	1.55
*Pleurotus citrinopileatus*	After 1st Centrifugation	84	378.74	0.83	69.88	184.52	100	1
	After 2nd Centrifugation	76	352.80	0.91	69.38	196.67	99.29	1.0
	Ammonium sulphate precipitation	4	10.95	0.93	3.73	341.15	5.34	1.84
	Dialysis	2	5.41	0.83	1.65	305.80	2.36	1.65
*Pleurotus florida*	After 1st Centrifugation	81.5	378.81	0.92	74.73	197.29	100	1
	After 2nd Centrifugation	78	363.84	1.04	71.21	195.92	95.28	0.99
	Ammonium sulphate precipitation	4	10.95	0.99	3.95	362.19	5.29	1.83
	Dialysis	2	5.56	1.01	2.01	362.30	2.69	1.83
*Hypsizygus ulmarius*	After 1st Centrifugation	84	390.77	0.96	80.38	205.71	100	1
	After 2nd Centrifugation	78.5	383.41	0.99	77.63	202.49	96.58	0.98
	Ammonium sulphate precipitation	4	11.11	0.99	3.94	355.28	4.91	1.72
	Dialysis	2	5.35	1.01	2.01	376.85	2.51	1.83
*Oudemansiella radicata*	After 1st Centrifugation	84	390.64	0.89	75.18	192.45	100	1
	After 2nd Centrifugation	76	354.94	0.93	70.27	198.27	93.61	1.03
	Ammonium sulphate precipitation	4	10.97	1.01	4.02	367.11	5.35	1.90
	Dialysis	2	5.35	0.98	1.95	366.20	2.60	1.90
*Volvariella volvacea*	After 1st Centrifugation	84.5	390.65	0.83	70.30	179.96	100	1
	After 2nd Centrifugation	81	380.08	0.96	70.22	184.76	99.89	1.02
	Ammonium sulphate precipitation	4	10.90	1.01	4.027	369.46	5.72	2.05
	Dialysis	2	5.31	0.99	1.97	372.50	2.81	2.06
*Schizophyllum commune*	After 1st Centrifugation	84	370.43	0.88	73.83	199.32	100	1
	After 2nd Centrifugation	83.5	389.95	0.89	70.30	100.29	95.22	0.90
	Ammonium sulphate precipitation	4	11.00	1.00	1.59	145.26	2.16	0.72
	Dialysis	2	5.21	0.94	1.88	360.92	2.54	1.81
*Tricholomopsis giganteus*	After 1st Centrifugation	84	390.43	0.85	71.48	183.09	100	1
	After 2nd Centrifugation	78.5	356.80	0.89	70.25	196.91	98.28	1.07
	Ammonium sulphate precipitation	4	10.93	0.98	3.93	359.63	5.49	1.96
	Dialysis	2	5.26	0.92	1.84	350.68	2.58	1.91

**Table 7:** Partial purification of Lignin Peroxidase from selected mushroom fungi (Results are mean of three replicates with SE < 5%).

Organisms	Purification steps	Volume (ml)	Total protein (mg)	Enzyme activity (×10^−5^ IU/ml)	Total activity (×10^−5^ IU)	Specific activity (×10^−5^ IU/mg)	Yield (%)	Purification (fold)
*Pleurotus djamor*	After 1st Centrifugation	84	387.80	0.92	77.28	199.27	100	1
	After 2nd Centrifugation	80	374.26	0.98	78.96	210.97	102.17	1.05
	Ammonium sulphate precipitation	4	10.99	0.92	3.69	336.16	4.7	1.68
	Dialysis	2	6.70	0.91	1.83	274.53	2.3	1.37
*Pleurotus citrinopileatus*	After 1st Centrifugation	84	378.74	0.77	65.18	172.10	100	1
	After 2nd Centrifugation	76	352.80	0.98	74.78	211.97	114.72	1.23
	Ammonium sulphate precipitation	4	10.95	0.88	3.53	222.84	5.42	1.87
	Dialysis	2	5.40	0.69	1.39	258.51	2.14	1.50
*Pleurotus florida*	After 1st Centrifugation	81.5	378.81	1.00	81.5	215.14	100	1
	After 2nd Centrifugation	78	363.84	0.88	69.10	289.94	84.78	0.88
	Ammonium sulphate precipitation	4	10.95	0.91	3.66	334.46	4.49	1.55
	Dialysis	2	5.56	0.93	1.86	334.92	2.28	1.55
*Hypsizygus ulmarius*	After 1st Centrifugation	84	390.77	0.77	65.18	165.53	100	1
	After 2nd Centrifugation	78.5	383.41	0.85	67.35	175.66	103.32	1.06
	Ammonium sulphate precipitation	4	11.11	0.89	3.55	320.90	5.44	1.93
	Dialysis	2	5.35	0.94	1.89	350.57	2.89	2.13
*Oudemansiella radicata*	After 1st Centrifugation	84	390.64	0.74	62.66	160.41	100	1
	After 2nd Centrifugation	76	354.94	1.02	77.74	219.04	124.07	1.36
	Ammonium sulphate precipitation	4	10.97	0.89	3.57	325.79	5.70	2.03
	Dialysis	2	5.35	0.85	1.71	320.11	2.75	1.98
*Volvariella volvacea*	After 1st Centrifugation	84.5	390.65	0.82	69.37	177.58	100	1
	After 2nd Centrifugation	81	380.08	0.96	78.48	206.50	113.13	1.16
	Ammonium sulphate precipitation	4	10.90	0.88	3.54	325.35	5.11	1.83
	Dialysis	2	5.31	0.90	1.81	341.24	2.61	1.92
*Schizophyllum commune*	After 1st Centrifugation	84	370.43	1.05	88.87	239.91	100	1
	After 2nd Centrifugation	83.5	389.95	0.81	68.21	174.94	76.76	0.73
	Ammonium sulphate precipitation	4	11.00	0.88	3.53	321.56	3.98	1.34
	Dialysis	2	5.21	0.86	1.73	332.09	1.94	1.38
*Tricholomopsis giganteus*	After 1st Centrifugation	84	390.43	0.97	81.48	208.69	100	1
	After 2nd Centrifugation	78.5	356.80	0.98	77.47	217.15	95.09	1.04
	Ammonium sulphate precipitation	4	10.93	0.87	3.51	320.00	4.31	1.53
	Dialysis	2	5.26	0.88	1.77	336.95	2.17	1.61

**Table 8:** Partial purification of Manganese Peroxidase from selected mushroom fungi (Results are mean of three replicates with SE < 5%).

Organisms	Purification steps	Volume (ml)	Total protein (mg)	Enzyme activity (×10^−5^ IU/ml)	Total activity (×10^−5^ IU)	Specific activity (×10^−5^ IU/mg)	Yield (%)	Purification (fold)
*Pleurotus djamor*	After 1st Centrifugation	84	387.80	1.61	135.40	349.16	100	1
	After 2nd Centrifugation	80	374.26	1.66	133.12	355.68	98.31	1.01
	Ammonium sulphate precipitation	4	10.99	1.72	6.86	624.56	5.06	1.78
	Dialysis	2	6.70	1.36	2.72	406.68	2.01	1.16
*Pleurotus citrinopileatus*	After 1st Centrifugation	84	378.74	1.77	148.80	393.00	100	1
	After 2nd Centrifugation	76	352.80	1.63	124	351.78	83.33	0.89
	Ammonium sulphate precipitation	4	10.95	1.63	6.52	595.90	4.38	1.51
	Dialysis	2	5.40	1.34	2.67	494.55	1.79	1.25
*Pleurotus florida*	After 1st Centrifugation	81.5	378.81	1.82	148	391.13	100	1
	After 2nd Centrifugation	78	360.84	1.72	133	370.93	89.89	0.94
	Ammonium sulphate precipitation	4	10.95	1.78	7.1	649.20	4.79	1.66
	Dialysis	2	5.56	1.42	2.8	511.65	1.89	1.30
*Hypsizygus ulmarius*	After 1st Centrifugation	84	390.77	1.811	151.70	388.21	100	1
	After 2nd Centrifugation	78.5	383.41	1.66	130.60	340.69	86.09	0.87
	Ammonium sulphate precipitation	4	11.11	1.73	6.91	621.99	4.55	1.60
	Dialysis	2	5.35	1.42	2.83	529.34	1.86	1.36
*Oudemansiella radicata*	After 1st Centrifugation	84	390.64	1.66	139	355.87	100	1
	After 2nd Centrifugation	76	354.94	1.76	133	376.82	95.68	1.05
	Ammonium sulphate precipitation	4	10.97	1.67	6.66	607.18	4.79	1.70
	Dialysis	2	5.35	1.36	1.35	253.45	0.97	0.71
*Volvariella volvacea*	After 1st Centrifugation	84.5	390.65	1.81	153.10	391.94	100	1
	After 2nd Centrifugation	81	380.08	1.77	143	376.35	93.46	0.96
	Ammonium sulphate precipitation	4	10.90	1.64	6.5	596.40	4.2	1.52
	Dialysis	2	5.31	1.32	2.64	497.19	1.72	1.26
*Schizophyllum commune*	After 1st Centrifugation	84	370.43	1.29	108.5	292.97	100	1
	After 2nd Centrifugation	83.5	389.95	1.31	109.3	280.51	100.73	0.95
	Ammonium sulphate precipitation	4	11.00	1.69	6.749	613.56	6.21	2.09
	Dialysis	2	5.21	1.36	2.71	520.99	2.49	1.77
*Tricholomopsis giganteus*	After 1st Centrifugation	84	390.43	1.70	143	366.61	100	1
	After 2nd Centrifugation	78.5	356.80	1.64	128.90	361.47	90.13	0.98
	Ammonium sulphate precipitation	4	10.93	1.65	6.59	603.55	4.60	1.64
	Dialysis	2	5.26	1.35	2.7	514.63	1.88	1.40

#### 3.6.1 Molecular weight determination of ligninolytic enzymes

**Fig. 2:**
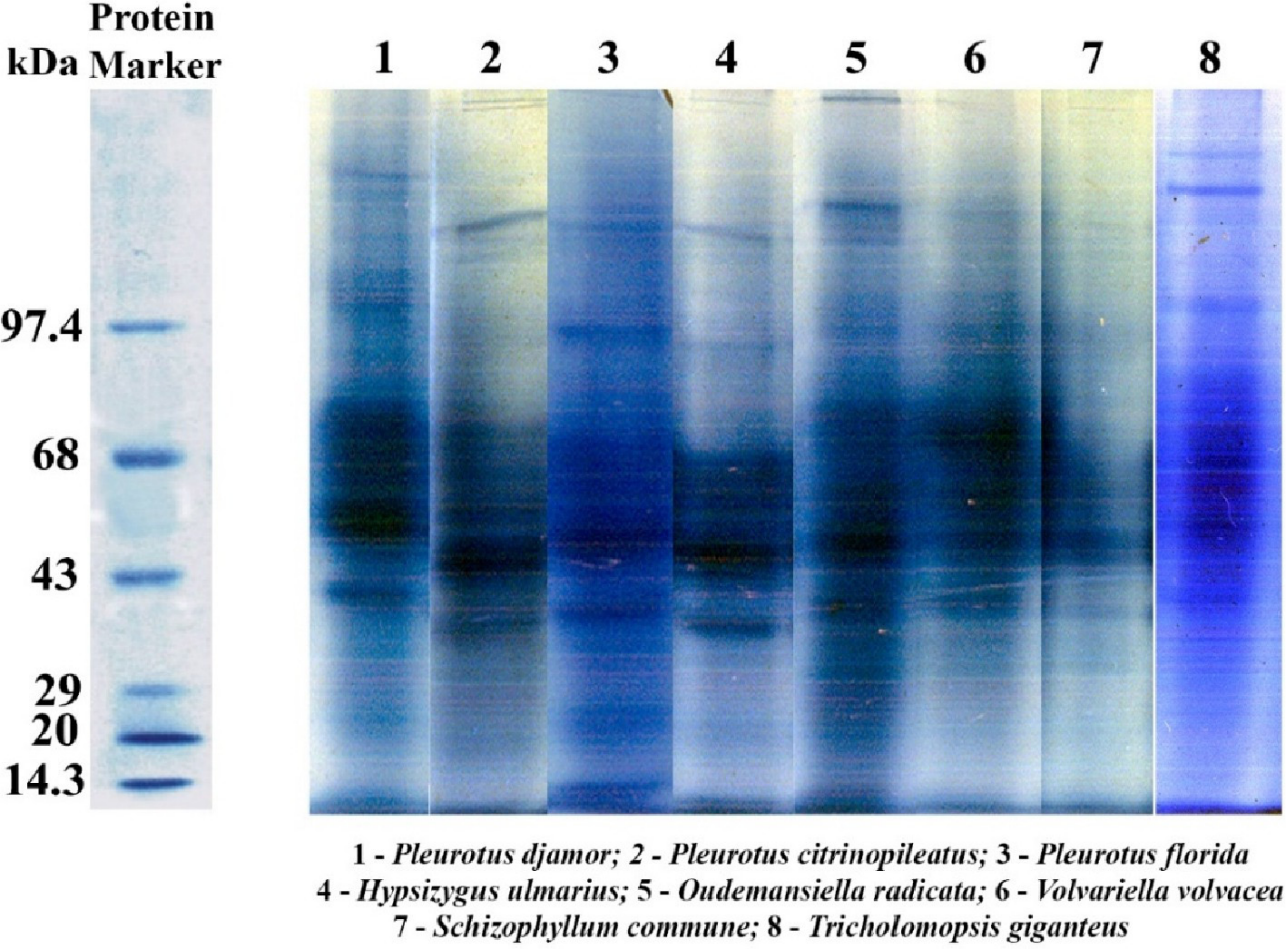
Mycelial protein profile in Native-PAGE of the selected basidiomycetes mushroom fungi.

**Fig. 3:**
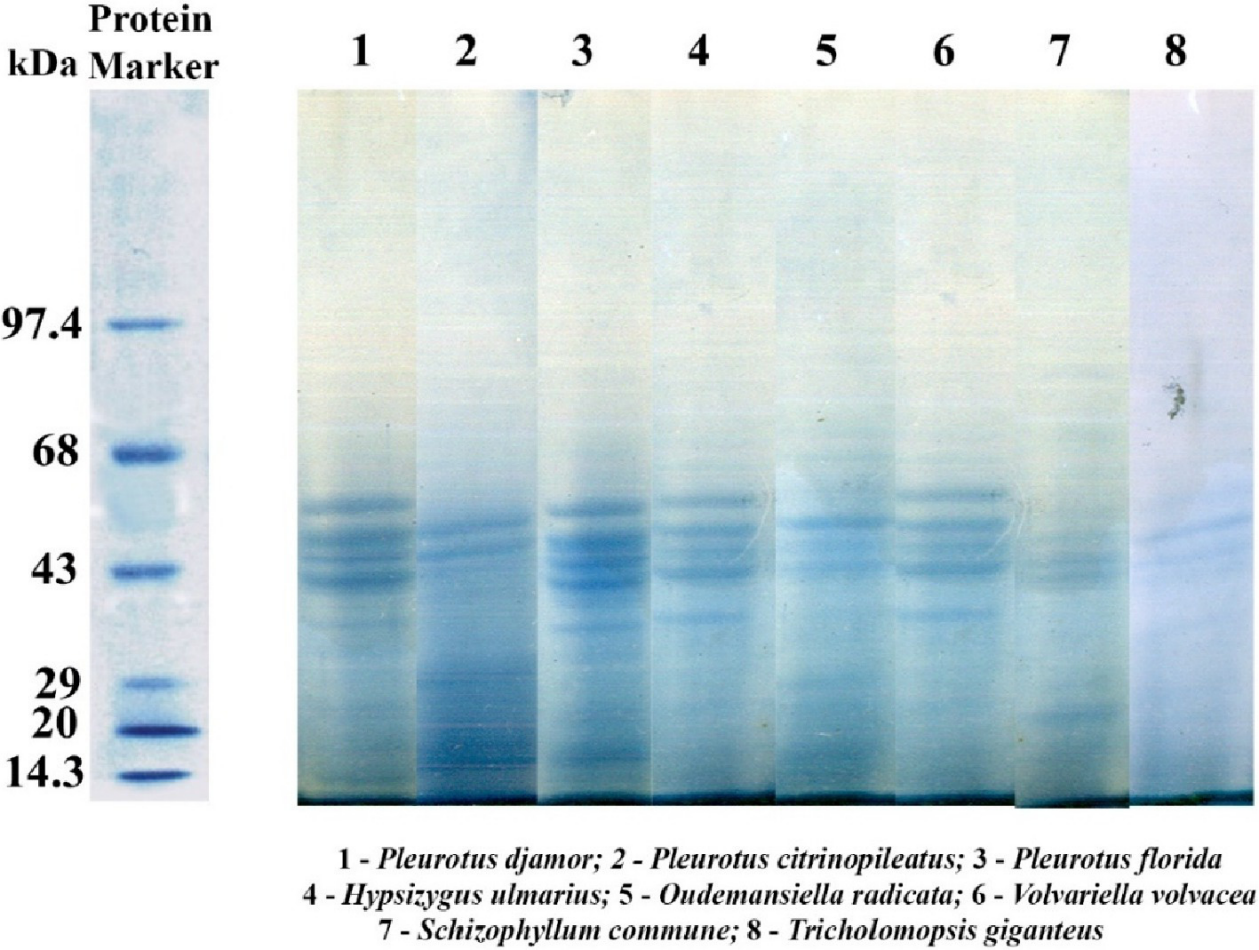
Mycelial protein profile in SDS-PAGE of the selected basidiomycetes mushroom fungi.

The homogeneity and molecular weight of the isolated ligninolytic enzymes were determined using native and SDS-PAGE. After electrophoresis on a native PAGE gel, mycelial proteins from the eight mushroom mushrooms were separated into three significant bands exhibiting molecular weights ranging from 43 to 68 kDa, and a single significant band with a molecular weight of 97.4 kDa (Fig. 2). At molecular weights between 68 and 97.4 kDa, and above MrS 97.4 kDa, many diffuse bands were visible. The SDS PAGE profiles of the eight mushroom fungus mycelial proteins were identical to the native PAGE. The molecular weights of the prominent bands in the centre of the gel ranged from 43 to 68 kDa. At MrS of 43 kDa, 55 kDa, and 68 kDa, SDS PAGE protein profiles revealed only one single band for each of the three prominent bands observed in native PAGE gels (Fig. 3).

**Fig. 4:**
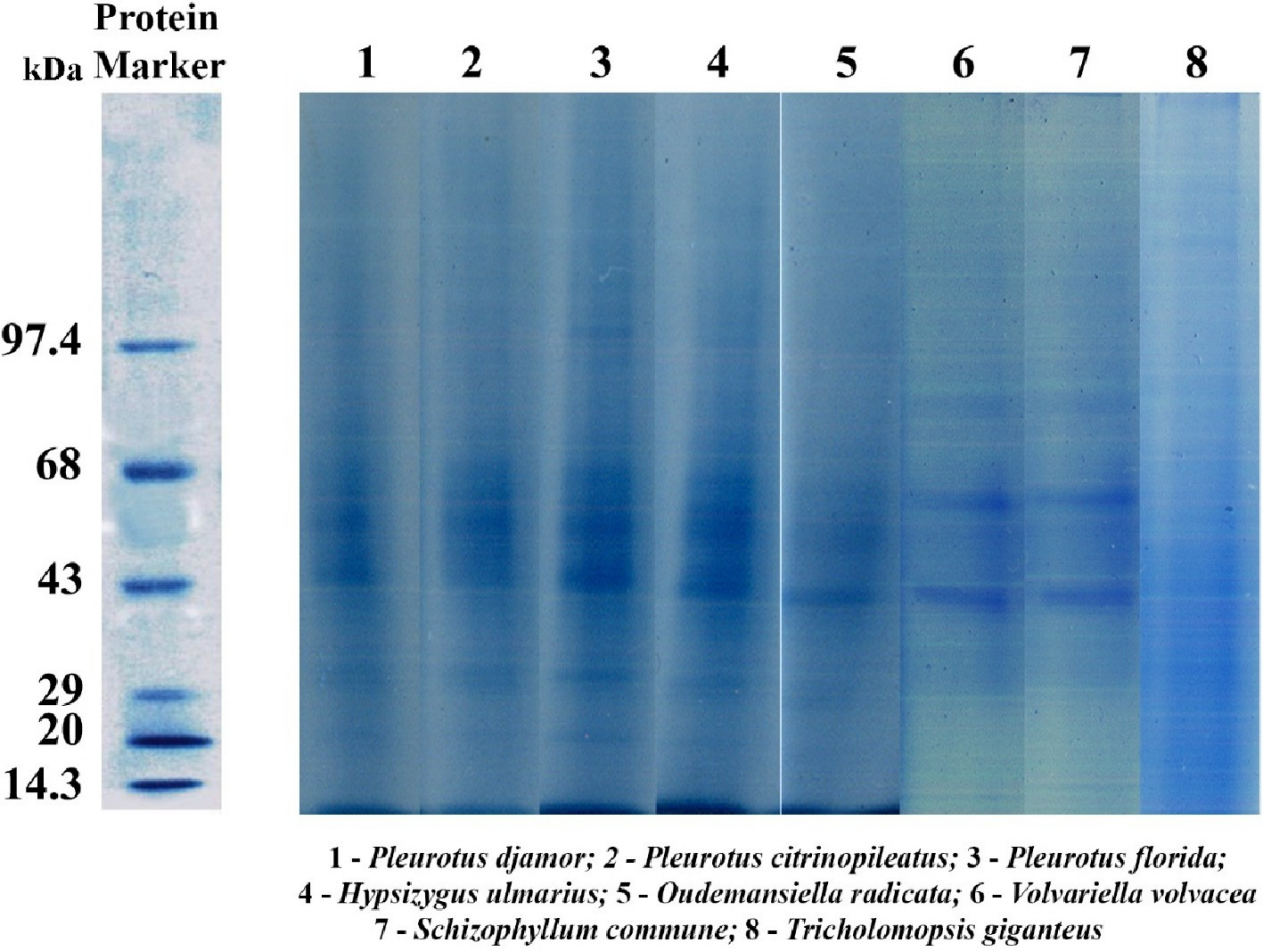
Partially purified extracellular ligninolytic enzyme profile of the selected mushroom fungi using Commassie brilliant blue stained SDS PAGE.

**Fig. 5:**
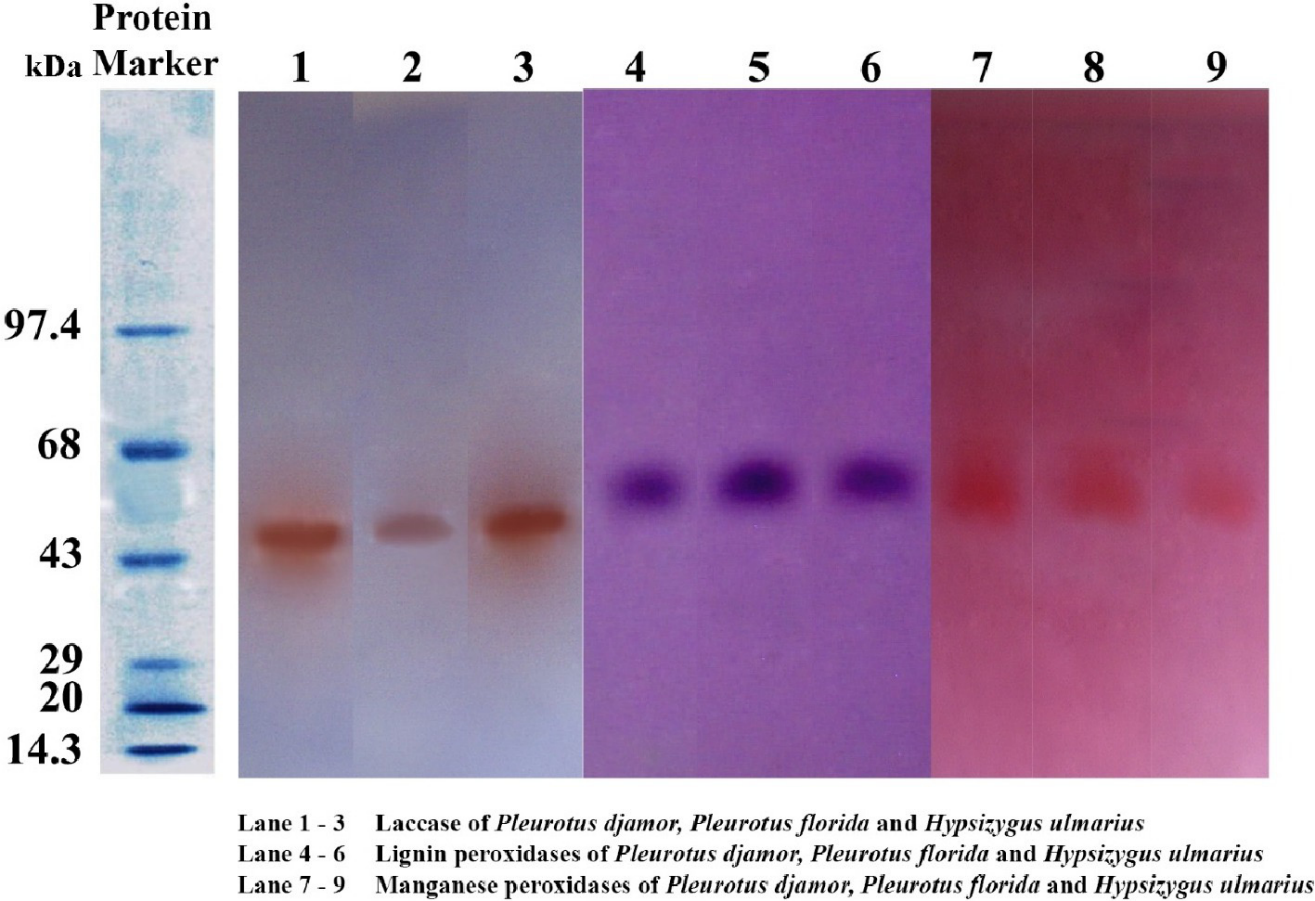
Partially purified extracellular ligninolytic enzyme profile of the selected mushroom fungi using enzyme stained native gel.

The purified extracellular ligninolytic enzyme profile of the selected mushroom fungi was found to have less bands on SDS PAGE than the mycelial protein profiles. However, at molecular weights between 43 and 68 kDa, three prominent bands with medium mobility were evident (Fig. 4). After staining with guiacol, enzyme activity staining of the purified extracellular ligninolytic enzyme showed that the 43 kDa protein band was a laccase, as it turned a reddish-brown colour. The protein band at 55 kDa stained purple violet when placed in a buffer containing veratryl alcohol, indicating that it is lignin peroxidase. The 68 k Da protein band became pinkish orange after staining with sodium tartrate buffer containing phenol red and MnSO_4_, indicating that it was manganese peroxidase (Fig. 5).

## 4 Discussion

The synthesis, purification, and characterisation of ligninolytic enzymes from edible basidiomycetes mushroom fungi are the focus of this research. Ligninolytic enzymes (Lac, LiP, MnP, and versatile peroxidase) are important for lignocellulosic waste decomposition and detoxification in the environment (Kumar and Chandra, 2020). These enzymes have attracted a lot of interest as biological agents for degrading lignocellulosic waste-containing substances as well as other organic contaminants. It has also been shown that ligninolytic enzymes are capable of decolorizing and biodegrading waste materials and other toxic compounds (Janusz et al., 2017, Shi et al., 2013).

Since increased biomass yield is essential for the economy of industrial processes based on mushroom mycelial production in fermentors for recovery of mushroom products, the growth rate, colonization, biomass production and the quantification of mycelial protein were used as parameters for screening the different mushroom genera. According to the findings of this study, *S. commune* and *H. ulmarius* were significantly efficient in the production of mycelial biomass, while *H. ulmarius* and *P. eous* had higher mycelial protein content, indicating their ability to be used in the commercial production of ligninolytic enzymes. Submerged culture of *Pleurotus* species like as *P. ostreatus, P. citrinopileatus*, and *P. florida* has been studied for the creation of liquid inoculums, extracellular enzymes, and antimicrobials by several authors (Chen et al., 2017, Ergun and Urek, 2017, Arpinar and Urek, 2014)*.*

White rot basidiomycetes and their enzymes could be used to convert lignocellulosics into a variety of products. Ligninolytic enzymes are produced by *Pleurotus* species, and they digest the primary component of lignocellulolytic biomass into a low molecular weight molecule that fungi may consume. Regulation of enzyme synthesis in lignocellulosic bioconversion could improve in the scientific advances of edible mushroom production or industrial biomass production. Since the ability of mushroom genera to produce ligninolytic enzymes is an indicator of their efficiency in substrate utilisation, ligninolytic enzyme plate assays and ligninolytic enzyme activities were used to screen the various mushroom genera. Similar observations had previously been made (Zheng et al., 2017, He et al., 2015, Akpinar and Urek, 2014, Masutti et al., 2014).

LiP, MnP, and laccase are the chief enzymes of white rot fungi to degrade lignin. Comprehensive research on basidiomycetous mushrooms has recently been carried out with the goal of isolating novel species with high production of ligninolytic and other enzymes with features critical for industrial application (Zhuo and Fan, 2021, Saldarriaga-Hernández et al., 2020, Zhang et al., 2020, Chandra and Madakka, 2019, Rodríguez-Couto, 2019, Cruz-Ornelas et al., 2019, Seppälä et al., 2017, Bilal et al., 2017, Kirsch et al., 2016).

The highest extracellular ligninolytic enzyme activities were observed on the 21st day in this study. *H. ulmarius* had substantially higher activities of all three ligninolytic enzymes, laccase (14.83 × 10^−6^ IU/ml), manganese peroxidase (24.11 × 10^−6^ IU/ml), and lignin peroxidase (10.57 × 10^−6^ IU/ml) among the fungi experimented in this research. Following this, *P. djamor* and *P. florida* had substantially higher ligninolytic enzyme activities.

Ammonium sulphate was used to concentrate the crude extract generated by all eight fungus. The infusion of ammonium sulphate to the protein solution absorbed water molecules that had previously coated the protein surfaces, enabling every protein to rest at the appropriate ammonium sulphate saturation point. The presence of ammonium sulphate attracts water surrounded in the hydrophobic region, permitting enzyme molecules to aggregate and precipitate (Fithri et al., 2020). The partial purification process includes the precipitation with ammonium sulphate. Ammonium sulphate showed higher concentration of ligninolytic enzymes at the optimal saturation percentage.

Table 6, Table 7, Table 8 demonstrate the results of partial purification of ligninolytic enzymes. *P. djamor* had a high specific activity of 382.52 × 10^−5^ IU/mg in laccase after precipitation with ammonium sulphate and a purification fold of 1.88, while the same strain had a specific activity of 336.16 × 10^−5^ IU/mg in lignin peroxidase and a purification fold of 1.68. For manganese peroxidase, *P. florida* had a maximum specific activity of 649.20 × 10^−5^ IU/mg with 1.66 fold purification. As specific activity improved from the culture supernatant to ammonium sulphate precipitation, the ligninolytic enzyme protein have further precipitated and might be separate from contaminating protein in crude extracts. Because the total protein following ammonium sulphate precipitation and dialysis was lower than the total protein in crude extract, the specific activity was higher (Zheng et al., 2017, Kumar et al., 2016).

Because the enzyme solution was devoid of ammonium sulphate salts as well as other proteins being disintegrated in the culture extract after dialysis, the specific activity of ligninolytic enzymes increased. Laccase, lignin peroxidases, and manganese peroxidases all had higher specific activity in *H. ulmarius*, with 376.85 × 10^−5^ IU/mg, 350.57 × 10^−5^ IU/mg, and 529.34 × 10^−5^ IU/mg respectively. The purification amount of ligninolytic enzyme obtained by ammonium sulphate salt precipitation and dialysis of laccase, lignin peroxidases, and manganese peroxidases from *H. ulmarius* crude extract was 1.83, 2.13, and 1.36 fold, respectively. The ammonium sulphate salt that had accumulated with the protein and enzyme was removed using dialysis. Proteins are separated from smaller ammonium sulphate molecules via a dialysis procedure. The importance of screening ligninolytic organisms for mass production and enzyme recovery in basidiomycetes mushroom fungi can be exploited for their enzymes is highlighted by the growing evidence for the industrial application and lignin degrading potential of ligninolytic enzymes (Pandey et al., 2018, Chen et al., 2017, Ire and Ahuekwe, 2016, Castaño et al., 2015).

The major components of the culture filtrates of the selected mushroom strains had molecular weights of 62 kDa, 55 kDa, and 45 kDa, respectively, which corresponds to the work of Zainith et al., 2000, Sharma et al., 2013. On SDS PAGE, each protein appeared as a separate band. In the SDS PAGE profiles of the culture filtrates of the selected mushroom genera, the number of extracellular enzyme proteins bands was less than the number of mycelial protein bands. The behaviour associations of ligninolytic enzymes with biomass are species specific and behave independently (Fonseca et al., 2013). In native gel electrophoresis, all three partially filtered ligninolytic isozymes display three bands, accompanied by only one single prominent band in enzyme activity staining, which is in line with prior research (Gu et al., 2014, Liu et al., 2009)

The potential applications of microbial ligninolytic enzymes in a extensive variation of commercial and other biological processes are being researched in a demanding, concentrated, and challenging manner. Xenobiotic or recalcitrant organic pollutants generated by a range of industries, as well as water management, effluent decomposition, and soil treatment, all require ligninolytic enzymes (Baruah et al., 2018, Su et al., 2018). Lignocellulosic product delignification, biopulping, biobleaching, denim cleaning, oil reserve depletion, and the converting of high-molecular-weight molecules to low-molecular-weight components are all viable commercial applications for this group of enzymes (Kumar and Chandra, 2020, Andriani et al., 2019).

## 5 Conclusion

Ligninolytic enzymes are a novel and hopeful method to industrial chemical process replacement. The natural habitat of fungi on Earth, which produces a range of organisms with the catalytic ability to execute lignocellulosic biomass hydrolysis, is a highly encouraged task as well as an ecologically responsible alternative for revalorization of agricultural and industrial waste using lignocellulolytic materials. Understanding the genetic pathways involved in the expansion of lignin-degrading enzymes is essential for completely understanding the lignin-degrading capacities of microbes. Improved saccharification of lignocellulosic biomass through enzyme hydrolysis requires the implementation of novel approaches, notably genetic engineering of fungi to boost the output of enzyme synthesis in a cost-effective manner, and also genetic expression predictions. Simultaneously, it's critical to investigate the structural and functional aspects of these ligninolytic enzymes, which can be done through a range of genetic engineering strategies.

## Declaration of Competing Interest

The authors declare that they have no known competing financial interests or personal relationships that could have appeared to influence the work reported in this paper.
